# Risk of stomach cancer in Aotearoa/New Zealand: A Māori population based case-control study

**DOI:** 10.1371/journal.pone.0181581

**Published:** 2017-07-21

**Authors:** Lis Ellison-Loschmann, Andrew Sporle, Marine Corbin, Soo Cheng, Pauline Harawira, Michelle Gray, Tracey Whaanga, Parry Guilford, Jonathan Koea, Neil Pearce

**Affiliations:** 1 Centre for Public Health Research, Massey University, Wellington, New Zealand; 2 Department of Statistics, The University of Auckland, Auckland, New Zealand; 3 Kimihauora Health Centre, Tauranga, New Zealand; 4 Centre for Translational Research, University of Otago, Dunedin, New Zealand; 5 Waitemata District Health Board, Auckland, New Zealand; 6 Department of Medical Statistics, London School of Hygiene & Tropical Medicine, London, England; Ruhr-Universitat Bochum, GERMANY

## Abstract

Māori, the indigenous people of New Zealand, experience disproportionate rates of stomach cancer, compared to non-Māori. The overall aim of the study was to better understand the reasons for the considerable excess of stomach cancer in Māori and to identify priorities for prevention. Māori stomach cancer cases from the New Zealand Cancer Registry between 1 February 2009 and 31 October 2013 and Māori controls, randomly selected from the New Zealand electoral roll were matched by 5-year age bands to cases. Logistic regression was used to estimate odd ratios (OR) and 95% confidence intervals (CI) between exposures and stomach cancer risk. Post-stratification weighting of controls was used to account for differential non-response by deprivation category. The study comprised 165 cases and 480 controls. Nearly half (47.9%) of cases were of the diffuse subtype. There were differences in the distribution of risk factors between cases and controls. Of interest were the strong relationships seen with increased stomach risk and having >2 people sharing a bedroom in childhood (OR 3.30, 95%CI 1.95–5.59), testing for *H pylori* (OR 12.17, 95%CI 6.15–24.08), being an ex-smoker (OR 2.26, 95%CI 1.44–3.54) and exposure to environmental tobacco smoke in adulthood (OR 3.29, 95%CI 1.94–5.59). Some results were attenuated following post-stratification weighting. This is the first national study of stomach cancer in any indigenous population and the first Māori-only population-based study of stomach cancer undertaken in New Zealand. We emphasize caution in interpreting the findings given the possibility of selection bias. Population-level strategies to reduce the incidence of stomach cancer in Māori include expanding measures to screen and treat those infected with *H pylori* and a continued policy focus on reducing tobacco consumption and uptake.

## Introduction

Although stomach cancer has declined in incidence over the past 4–5 decades, it remains an important public health problem and is still one of the most common cancers worldwide [1]. Stomach cancer has one of the largest ethnic inequalities of any cancer site in Aotearoa/New Zealand (hereafter referred to as NZ) with rates in Māori, the indigenous population, being up to five times those of non-Māori in the late 1990s [2] and is currently one of the major five cancers contributing to NZ’s ethnic gap in cancer incidence alongside lung, breast, liver and endometrial cancers [3]. The most recent published data available from the NZ Cancer Registry (NZCR) for 2012 indicate Māori registration rates to be more than three times those of non-Māori (15.8 vs 4.8 per 100,000 respectively) and death rates 3.7 times higher in Māori compared to non-Māori (13.1 vs 3.5 per 100,000 respectively) [4]. Māori comprise almost 15% of the total 4.5 million population of NZ. The other major ethnic groupings are Pasifika (7%), Asian (12%) and European/Other peoples (from primarily the United Kingdom and Europe) (74%) [5].

Approximately 95% of stomach cancers are adenocarcinomas, divided histologically into two major subtypes: intestinal and diffuse [6]. Declines in stomach cancer incidence over the past forty years have occurred primarily for the intestinal type [7]. Māori appear to develop stomach cancer at a younger age and have a higher incidence of diffuse adenocarcinomas compared to non-Māori [8–9]. Diffuse tumours are seen in hereditary diffuse gastric cancer which is associated with germline E-cadherin gene (CDH1) alterations [10] however reasons for the higher incidence of sporadic diffuse cancers in Māori remain unclear.

Stomach cancer may arise in the proximal (cardia) or distal (non-cardia) body of the stomach or the oesophago-gastric (OG) junction. Development of distal stomach cancer is associated with chronic exposure to *Helicobacter pylori* (*H pylori*), a common bacterium in the stomach lining which is usually acquired in childhood [11–12]. Higher rates of distal stomach cancer have been reported in Māori compared to non-Māori [9] with over half of the excess distal stomach cancer incidence in Māori men estimated to be due to infection by *H pylori* [13]. Household crowding is linked to *H pylori* infection [13–15] and in NZ, overcrowding is disproportionately experienced by Māori and Pasifika peoples [15]. Previous NZ studies undertaken during 1970-1980s documented stomach cancer mortality risk to be up to four times greater for the lowest compared to the highest social class grouping [16,17] with latest reported time trends showing a decrease in stomach cancer mortality for all income groups between 1981–84 and 2006–11 [18].

Obesity is a major contributing risk factor to the rapid increase in proximal and OG junction stomach cancers [19]. Rates of obesity in NZ have more than trebled since the 1970s with 2013/2014 data showing 46% of Māori compared to 30% of the total adult population are obese and that rates of doctor diagnosed diabetes in Māori are double those of non-Maori [20]. While diabetes has been documented as a risk factor for a number of cancers [21] few studies have investigated the relationship between diabetes and stomach cancer [22–23].

Dietary factors, specifically, low intake of fruit and vegetables and high intake of salty foods and processed meat, have been found to be associated with an increased risk of stomach cancer [24–26]. Positive associations between tobacco smoking and stomach cancer have been reported [27–28]. Māori have had some of the highest smoking rates in the world and begin smoking at an earlier age than other populations [29–30]. An association with alcohol consumption [25] and stomach cancer risk has also been found while the evidence for physical activity and stomach cancer varies according to intensity of activity and cancer site [31].

Thus, while there is some evidence of higher exposures in Māori for some of these risk factors there is insufficient information to quantify their relative importance, or to ascertain the role of other potential risk factors. The overall aim of the study was to better understand the reasons for the considerable excess of stomach cancer in Māori and to identify priorities for prevention. This paper presents the methods and findings in relation to established risk factors for stomach cancer in a Māori population-based case control study.

## Materials and methods

All Māori with a diagnosis of stomach cancer (ICD10 C16), based on histology reports sent to the NZ Cancer Registry (NZCR) between 1 February 2009 and 31 October 2013, were eligible for inclusion in the study. The NZCR is a population based register of all primary malignant diseases. Because of the time lapse for registration of stomach cancer (up to one year following diagnosis) we identified cases using histology reports for confirmation of diagnosis which were forwarded from the NZCR to the study team every month. Ethnicity was identified from the NZCR which assign ‘Māori’ as the ethnic group if the person has self-identified as Māori on a previous health record. The clinician (named on the histology report as either a specialist or general practitioner) was contacted via letter or phone call seeking permission to contact the patient, with up to 3 reminder letters faxed within a 2 week period. Each case was then contacted by post and followed up with two reminder letters.

Controls were block sampled from the NZ electoral roll based on the age of the cases in 5-year age bands. Registration on the electoral roll is compulsory for all people 18 years and over and is the most comprehensive and complete population sampling frame available in NZ. Māori can choose to register on either the ‘General’ or ‘Māori’ roll. All people on the General roll are asked to self-identify as to whether they are Māori or the descendent of a Māori. Māori controls were randomly chosen from both rolls (Māori—52.2%, General—47.8%) using the 2008, 2010 and 2012 electoral rolls. There were large numbers of non-responders, primarily due to high mobility among younger Māori [32]. We therefore used an additional database of Māori population-based controls from a recently completed study which had more up-to-date contact information. Controls sampled from this database included both those who had consented to take part in the earlier study and those who did not. Thus, all controls for the current study were selected from the same source population, the electoral roll.

### Exposure measurement

Participants were given the option of completing the questionnaire with a trained interviewer face-to-face, by telephone or returning it by post. A blood sample was also requested to test for: *H pylori* serology, heritable genetic alterations and polymorphisms, antioxidant levels, serum trace elements and heavy metal levels. This latter information is provided for completeness but blood analyses will be reported separately in future publications.

The NZ Deprivation Index 2006 (NZDep2006) [33] was used to measure socio-economic position (SEP) based on place of residence at the date of diagnosis for cases and date of interview for controls. The index uses data from the national Census (in this instance, 2006) to create a deprivation measure for each small geographic area. These area measures are then ranked and aggregated into deciles with approximately equal numbers of areas in each decile. Ten represents the most deprived 10% while ‘1’ represents the least deprived 10% of areas. For these analyses, deciles were combined 1–2, 3–4, 5–6, 7–8 and 9–10 and assessed in quintiles as Q1, Q2, Q3, Q4 and Q5 respectively.

Where possible, validated questions were used [34]. Age was defined as age at diagnosis for the cases and age at interview for the controls. The questionnaire comprised sections on childhood socio-demographics, parent and sibling cancer history, occupational history, smoking (never, current, ex-smoker) and as an adult, living with someone who smoked regularly (yes/no). Responses to questions on number of rooms used for sleeping, i.e bedrooms and living room, and number of people living in the childhood home were used to create the variable ‘Childhood people to room ratio’ (< = 1.0/>1.0–2.0/>2.0) based on the definition of crowding used by Statistics NZ [35]. Both cases and controls were asked to report their lifestyles one year previously for: exercise frequency (none, 1–2, 3–4, 5+ times/week); alcohol intake (never, monthly or less, 2–4 times/month, 2–3 times/week, 4+ times/week); dietary intakes of red meat, white meat, fish and dried/salty food (times/week consumed–none, 1–2, 2–3, 3–4, 5+/week); servings of fruit (<7, 7–14, ≥14 /week) and vegetables (excluding potatoes) /week (<7, 7–21, ≥21) (using NZ recommended guidelines) [36]. Participants were also asked their weight and height (one year prior to diagnosis for cases) to determine body mass index (BMI). BMI was categorised into <25 kg/m^2^, 25–30 kg/m^2^ (overweight), >30 kg/m^2^ (obese). Questions were asked on diabetes (‘*have you ever been told by a doctor that you have diabetes or sugar in the blood*?’—yes/no), having ever been tested for *H pylori* (yes/no/don’t know) and doctor diagnosed dyspepsia (yes/no/don’t know).

### Statistical analysis

Descriptive statistics were used to explore the variable values and summarize the data. Chi-square tests were used to compare exposure distributions between cases and controls. Continuous variables were categorised using pre-defined cut-offs or quantiles. We used logistic regression (SAS V9.3) to estimate odds ratios (OR) and 95% confidence intervals (CI) adjusted for age and gender for each potential risk factor.

Because of the low response rates in the control group (see below) and the evidence of differential non-response by deprivation quintile, we performed a sensitivity analysis to investigate non-response bias, using post-stratification weights. A weight was calculated for each stratum of deprivation, by dividing the expected deprivation distribution by the observed deprivation distribution in the controls from our study. The expected distributions were estimated from the 2002/2003 NZ Health Survey [37] and were 2, 3, 10, 20, and 65% for Māori in quintiles 1–5 of the NZDep2006 categories. Logistic regression models were then weighted using the ‘WEIGHT’ statement of the SAS LOGISTIC procedure.

#### Ethical approval

The study was granted ethics approval by the NZ Multi-region Ethics Committee (ref: MEC/08/08/102/AM03). Informed written consent was obtained from all study participants.

## Results

**Fig 1:**
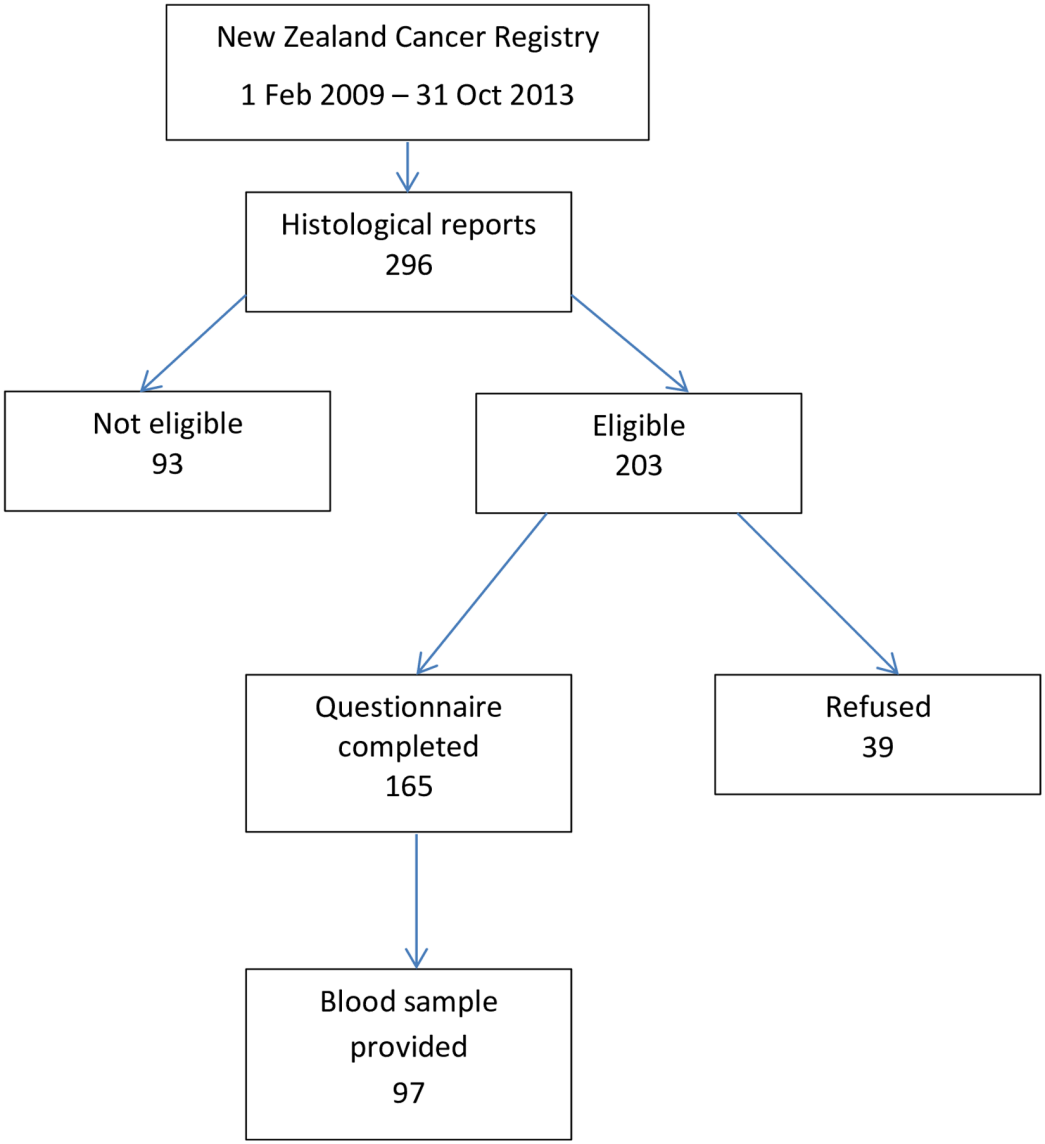
Flow diagram showing case participation.

A total of 296 Māori with stomach cancer were identified from histological reports sent to the NZCR for the 5 year time period of the study. Of these, 93 were ineligible to take part because they died before they could be contacted [*n* = 65], were too unwell [*n* = 24], did not have stomach cancer [*n* = 2] or were not Māori [*n* = 2]. Specifically, 63.7% (n = 79) of case non-participants had metastatic stage cancers at diagnosis compared to 36.4% (n = 60) of participants. Of the 203 eligible cases, 165 (81%) completed a questionnaire of which 97 (59%) also provided a blood sample (Fig 1).

There were no differences in median age (58 years) or gender between eligible case participants and non-participants; however, there were differences by NZDep2006 categories, particularly for Q3 (13% vs 5% respectively).

**Fig 2:**
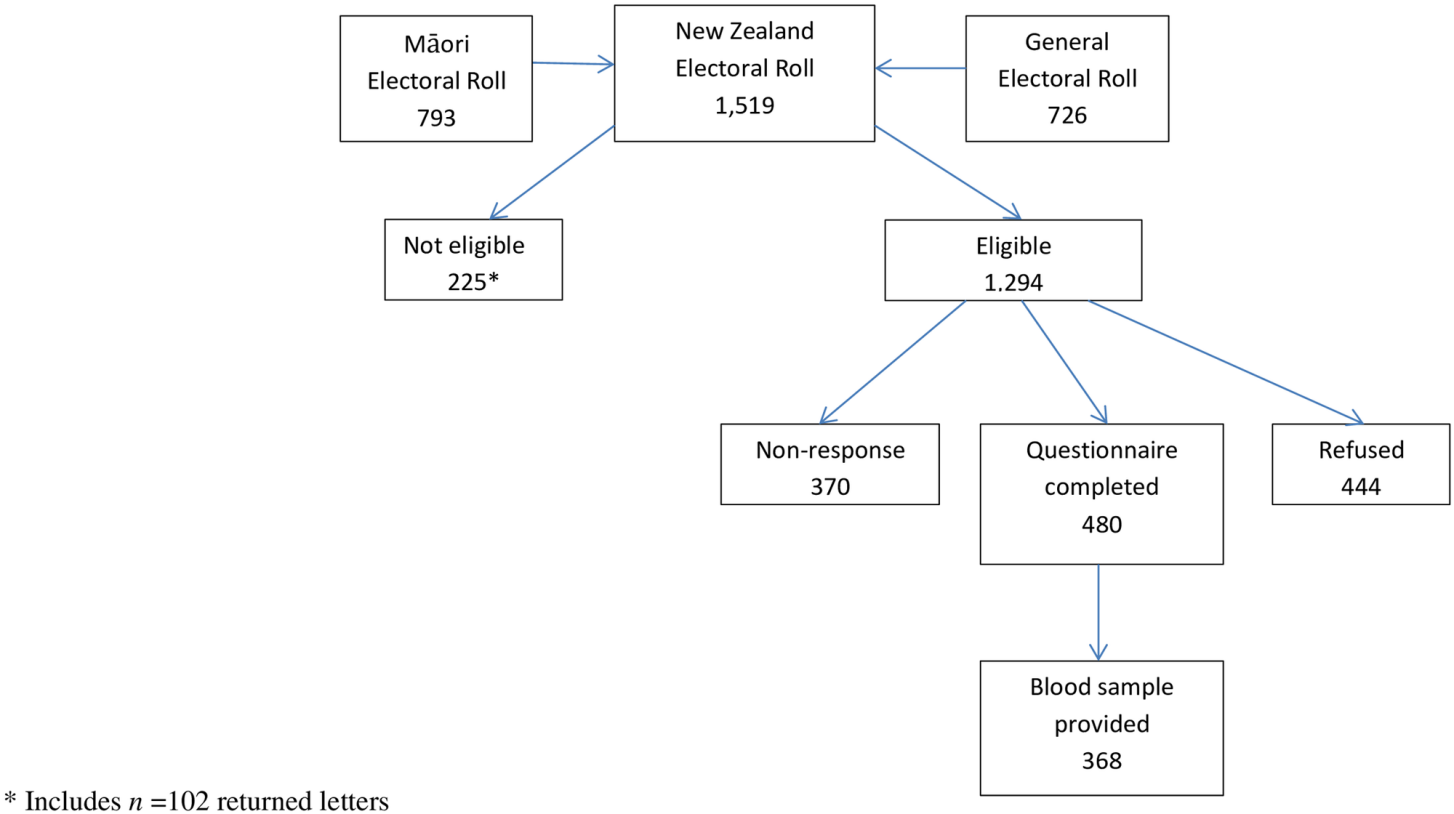
Flow diagram showing control participation.

A total of 1,519 controls were sampled comprising n = 726 (48%) from the General electoral roll and n = 793 (52%) from the Māori roll (Fig 2).

Of these, 102 (7%) were returned as undelivered and 123 (8%) were not eligible because they died before they could be contacted (n = 29), were no longer living in NZ (n = 28), were too ill to participate (n = 52), were not Māori (n = 11) or had a stomach cancer diagnosis (n = 3). Amongst the eligible population (*n* = 1,294), 480 (37%) people completed the questionnaire with 368 (77%) also providing a blood sample. Amongst eligible controls, participants were slightly older (median age 64 years [Inter quartile range [IQR] 53–77]) than non-participants (median age 60 years [IQR 46–75]) and were more likely to be from NZDep2006 quintile 4 and less likely to be from the most deprived quintile group (NZDep2006 Q5) than non-participants.

Table 1 shows the distribution of risk factors by cases and controls. There was a higher proportion of cases from the youngest age group (≤35 years) and the most deprived quintile, Q5, compared to controls. Controls were less likely to have lived in houses during their childhood where > 2 people shared a bedroom. They were also more likely to be non-smokers while the proportion of current smokers was similar in both cases and controls. Being regularly exposed to environmental tobacco smoke (ETS) as an adult was less common amongst controls. Fish and dried/salty foods consumption was lower amongst controls, as were the daily recommended intakes of fruit and vegetables (2 pieces/day and 3 servings/day respectively). Controls were however, more likely to have drunk alcohol in the previous year. A diagnosis of diabetes and dyspepsia was less common amongst controls as was reporting a family history of stomach cancer and having been tested for *H pylori* (2.9% vs 18.2% in cases).

**Table 1:** Distribution of known risk factors for stomach cancer in cases and controls.

	Total 645 (n)	%	Cases 165 (n)	%	Controls 480 (n)	%	p-value
**Gender**							
Male	328	50.8	84	50.9	244	50.8	
Female	317	49.2	81	49.1	236	49.2	0.987
**Age at interview or cancer diagnosis**							
< = 35	49	7.6	21	12.7	28	5.8	
>35–50	118	18.3	27	16.4	91	19.0	
>50–65	238	36.9	64	38.8	174	36.2	
>65	240	37.2	53	32.1	187	39.0	0.020
**Interview method**							
Phone	176	27.3	44	26.7	132	27.5	
Face	433	67.1	119	72.1	314	65.4	
Post	36	5.6	2	1.2	34	7.1	0.015
**Deprivation quintile (Q)**							
Q1 (least)	66	10.2	8	4.9	58	12.1	
Q2	89	13.8	11	6.7	78	16.2	
Q3	101	15.7	22	13.3	79	16.5	
Q4	167	25.9	38	23.0	129	26.9	
Q5 (most)	222	34.4	86	52.1	136	28.3	<0.0001
**Smoking status**							
Non-smoker	199	30.8	35	21.2	164	34.2	
Ex-smoker	301	46.7	93	56.4	208	43.3	
Current smoker	145	22.5	37	22.4	108	22.5	0.004
Exposure to ETS* **as an adult**							
Yes	489	75.8	147	89.1	342	71.3	
No	156	24.2	18	10.9	138	28.7	<0.0001
Childhood people to room ratio**							
< = 1.0	189	29.3	34	20.6	155	32.3	
>1.0–2.0	294	45.6	72	43.6	222	46.3	
>2.0	156	24.2	56	34.0	100	20.8	0.0006
Missing	6	0.9	3	1.8	3	0.6	
**Parent with stomach cancer**							
Yes	49	7.6	29	17.6	164	4.1	
No	553	85.7	126	76.3	208	89.0	<0.0001
Missing	43	6.7	10	6.1	108	6.9	
**BMI (kg/m**^**2**^**)**							
<25	185	28.7	69	41.8	116	24.2	
25–30	215	33.3	59	35.8	156	32.5	
>30	232	36.0	37	22.4	195	40.6	<0.0001
Missing	13	2.0	0	0.0	13	2.7	
**In the past year, how many times/week did you exercise?**							
None	165	25.6	42	25.4	123	25.6	
1–2	118	18.3	20	12.1	98	20.4	
3–4	135	20.9	41	24.9	94	19.6	
5+ / week	227	35.2	62	37.6	165	34.4	0.086
**In the past year, how many servings /week did you eat vegetables?**							
<7	127	19.7	22	13.3	105	21.9	
7–21	362	56.1	86	52.1	276	57.5	
> = 21 / week	156	24.2	57	34.6	99	20.6	0.0005
**In the past year, how many servings/week did you eat fruits?**							
<7	215	33.3	42	25.5	173	36.1	
7–14	182	28.2	49	29.7	133	27.7	
> = 14 / week	248	38.5	74	44.8	174	36.2	0.036
**In the past year, how many times/week did you eat red meat?**							
None	45	7.0	16	9.7	29	6.0	
1–2	179	27.8	45	27.3	134	27.9	
3–4	284	44.0	71	43.0	213	44.4	
5+ / week	137	21.2	33	20.0	104	21.7	0.461
**In the past year, how many times/week did you eat white meat?**							
None	33	5.1	11	6.7	22	4.6	
1–2	344	53.3	73	44.2	271	56.5	
3–4	236	36.6	75	45.5	161	33.5	
5+ / week	32	5.0	6	3.6	26	5.4	0.018
**In the past year, how many times/week did you eat fish?**							
None	161	25.0	31	18.8	130	27.1	
1–2	390	60.4	93	56.4	297	61.9	
3–4	76	11.8	33	20.0	43	8.9	
5+ / week	18	2.8	8	4.8	10	2.1	0.0002
**In the past year, how many times /week did you eat dried or salty food?**							
None	304	47.1	56	34.0	248	51.7	
1–2	280	43.4	82	49.7	198	41.2	
3–4	41	6.4	21	12.7	20	4.2	
5+ / week	20	3.1	6	3.6	14	2.9	<0.0001
**In the past year, how often did you have a drink containing alcohol?**							
Never	208	32.2	70	42.4	138	28.7	
Monthly or less	164	25.4	58	35.1	106	22.1	
2–4 times/month	99	15.4	16	9.7	83	17.3	
2–3 times/ week	82	12.7	10	6.1	72	15.0	
4 + times /week	92	14.3	11	6.7	81	16.9	<0.0001
**Ever diagnosed with diabetes**							
Yes	136	21.1	46	27.9	90	18.8	
No	509	78.9	119	72.1	390	81.2	0.013
**Ever tested for *H pylori***							
Yes	44	6.8	30	18.2	14	2.9	
No	514	79.7	75	45.4	439	91.5	<0.0001
Don't know	87	13.5	60	36.4	27	5.6	
**Ever diagnosed with dyspepsia**							
Yes	115	17.8	47	28.5	68	14.2	
No	517	80.2	110	66.7	407	84.8	<0.0001
Don't know	13	2.0	8	4.8	5	1.0	

* Environmental tobacco smoke

** Number of people living in house divided by number of rooms

Cancer details for cases are presented in Table 2. The diffuse subtype made up almost half of all cancers with 64.2% of women and 35.7% of men diagnosed with this subtype. For tumour grade, 43.0% were poorly differentiated with 38.8% recorded as unknown. The majority of cancers were recorded as late stage (36.4%) followed by unstaged (34.5%), of these; men were more likely to have unstaged cancer than women (40.5 vs 28.4% respectively). Men also had a higher proportion of tumours arising in the OG junction (10.7%) and proximal sites (34.5%) than women (1.3% and 29.6% respectively) while distal tumours were more common in women than men (29.6 vs 16.7%). Stomach with an unspecified anatomical site was the largest cancer group (33.3%).

**Table 2:** Tumour characteristics of stomach cancer case participants.

	Total 165 (n)	%	Males 84 (n)	%	Females 81 (n)	%	p-value
**Subtype**							
Intestinal	43	26.0	27	32.2	16	19.8	
Diffuse	82	49.7	30	35.7	52	64.2	
NOS	28	17.0	19	22.6	9	11.1	0.004
Other	12	7.3	8	9.5	4	4.9	
**Tumour grade**							
Well differentiated	8	4.9	4	4.8	4	4.9	
Moderately differentiated	22	13.3	13	15.5	9	11.1	
Poorly differentiated	71	43.0	37	44.0	34	42.0	0.789
Unknown	64	38.8	30	35.7	34	42.0	
**Tumour site**							
Oesophageal junction	10	6.1	9	10.7	1	1.3	
Proximal	53	32.1	29	34.5	24	29.6	
Distal	38	23.0	14	16.7	24	29.6	
Mixed	9	5.5	3	3.6	6	7.4	0.028
NOS	55	33.3	29	34.5	26	32.1	
**Stage**							
Local	37	22.4	17	20.2	20	24.7	
Regional	11	6.7	4	4.8	7	8.6	
Distant	60	36.4	29	34.5	31	38.3	0.362
Unstaged	57	34.5	34	40.5	23	28.4	

Associations between known risk factors and stomach cancer are shown in Table 3. Weighted results to account for differential non-response by deprivation category are also presented. Increasing deprivation was non-significantly associated with an elevated risk of stomach cancer except in the most deprived quintile Q5 (OR 4.72, 95% 2.14–10.41). Having >2 people sharing a bedroom in childhood (OR 3.30, 95%CI 1.95–5.59), being an ex-smoker (OR 2.26, 95%CI 1.44–3.54) and adult exposure to ETS (OR 3.29, 95%CI 1.94–5.59) were all associated with increased odds of stomach cancer. A non-significant increased odds ratio was also seen with current smoking (OR 1.46, 95%CI 0.86–2.48). Being tested for *H pylori* (OR 12.17, 95%CI 6.15–24.08), having diabetes (OR 2.03, 95%CI 1.32–3.14) or dyspepsia (OR 2.61, 95%CI 1.70–4.01) and having a parent with stomach cancer (OR 4.54, 95%CI 2.45–8.40) were all associated with statistically significant increased odds of stomach cancer. Increased frequency of eating dried/salty food and the highest categories (3–4 and 5+ times/week) of fish consumption were associated with an increased odds of stomach cancer, while a non-significant protective effect was observed for red and white meat consumption. An unexpected protective effect was seen with lower categories of fruit and vegetable intake. Protective effects were also observed for the highest categories of alcohol intake and for the 25–30 and >30 kg/m^2^ categories of BMI. No clear pattern was seen with stomach cancer risk and exercise frequency.

**Table 3:** Adjusted odds ratios (OR) and 95% confidence intervals (CI) showing the association between known risk factors and stomach cancer risk.

	Adjusted ORs ^a^ OR [95% CI]	Adjusted and weighted ORs ^b^ OR [95%CI]
**Demographics**		
**Deprivation quintile (Q)**		
Q1 (least)	1*	1*
Q2	1.06 [0.40–2.80]	0.94 [0.27–3.20]
Q3	2.10 [0.87–5.05]	0.56 [0.19–1.64]
Q4	2.20 [0.96–5.02]	0.48 [0.18–1.34]
Q5 (most)	4.72 [2.14–10.41]	0.34 [0.13–0.89]
Childhood people to room ratio**		
< = 1.0	1*	1*
>1.0–2.0	1.57 [0.99–2.50]	1.35 [0.84–2.16]
>2.0	3.30 [1.95–5.59]	1.91 [1.13–3.24]
**Parent with stomach cancer**		
No	1*	1*
Yes	4.54 [2.45–8.40]	3.93 [2.17–7.14]
**Lifestyle factors**		
**Smoking status**		
Non-smoker	1*	1*
Ex-smoker	2.26 [1.44–3.54]	1.80 [1.15–2.83]
Current smoker	1.46 [0.86–2.48]	1.01 [0.59–1.71]
Exposure to ETS*** **as an adult**		
No	1*	1*
Yes	3.29 [1.94–5.59]	2.58 [1.51–4.40]
**BMI (kg/m**^**2**^**)**		
<25	1*	1*
25–30	0.64 [0.42–0.98]	0.65 [0.42–01.01]
>30	0.32 [0.20–0.50]	0.27 [0.17–0.43]
**In the past year, how many times/week did you exercise?**		
None	1*	1*
1–2	0.55 [0.30–1.01]	0.73 [0.40–1.34]
3–4	1.15 [0.68–1.94]	1.39 [0.82–2.33]
5+ / week	0.99 [0.61–1.60]	1.11 [0.69–1.78]
**In the past year, how often did you have drink containing alcohol?**		
Never	1*	1*
Monthly or less	0.96 [0.62–1.50]	0.88 [0.57–1.36]
2 to 4 / month	0.31 [0.16–0.58]	0.38 [0.20–0.70]
2 to 3 / week	0.22 [0.11–0.47]	0.34 [0.16–0.70]
4 or / week	0.24 [0.12–0.48]	0.34 [0.17–0.70]
**Nutrition factors**		
**In the past year, how many servings /week did you eat vegetables?**		
<7	0.31 [0.18–0.56]	0.26 [0.14–0.46]
7–21	0.50 [0.33–0.75]	0.46 [0.30–0.70]
> = 21 / week	1*	1*
**In the past year, how many servings/week did you eat fruits?**		
<7	0.52 [0.33–0.81]	0.49 [0.32–0.76]
7–14	0.82 [0.53–1.26]	0.78 [0.51–1.20]
> = 14 / week	1*	1*
**In the past year, how many times/week did you eat red meat?**		
None	1*	1*
1–2	0.57 [0.28–1.16]	0.64 [0.32–1.29]
3–4	0.55 [0.28–1.08]	0.61 [0.31–1.19]
5+ / week	0.52 [0.25–1.09]	0.59 [0.28–1.24]
**In the past year, how many times/week did you eat white meat?**		
None	1*	1*
1–2	0.52 [0.24–1.12]	0.57 [0.26–1.21]
3–4	0.86 [0.40–1.88]	0.91 [0.42–1.97]
5+ / week	0.40 [0.12–1.26]	0.54 [0.17–1.74]
**In the past year, how many times/week did you eat fish?**		
None	1*	1*
1–2	1.56 [0.97–2.50]	1.70 [1.06–2.73]
3–4	3.77 [2.03–6.99]	3.75 [2.04–6.92]
5+ / week	4.52 [1.60–12.75]	4.41 [1.59–12.23]
**In the past year, how many times a week did you eat dried/salty food?**		
None	1*	1*
1–2	1.81 [1.23–2.67]	1.83 [1.24–2.70]
3–4	4.40 [2.23–8.70]	6.48 [3.10–13.57]
5+ / week	1.89 [0.69–5.16]	2.73 [0.93–8.01]
**Other health factors**		
**Ever diagnosed with diabetes**		
No	*1	*1
Yes	2.03 [1.32–3.14]	1.45 [0.95–2.20]
**Ever tested for *H pylori***		
No	*1	*1
Yes	12.17 [6.15–24.08]	9.02 [4.81–16.92]
**Ever diagnosed with dyspepsia**		
No	*1	*1
Yes	2.61 [1.70–4.01]	2.59 [1.69–3.98]

* Reference group

** Number of people living in house divided by number of rooms

*** Environmental tobacco smoke

^a^ OR adjusted for gender and age

^b^ OR adjusted for gender, age and weighted using post-stratification weights to account for differential non-response bias by deprivation quintile.

For the weighted analyses, an overall attenuation of the effect estimates was seen, but a statistically significant increased odds of stomach cancer remained for the associations with being an ex-smoker (OR 1.80, 95%CI 1.15–2.83), exposure to ETS (OR 2.58, 95%CI 1.51–4.40), > 2 people sharing a bedroom in childhood (OR 1.91, 95%CI 1.13–3.24), being tested for *H pylori* (OR 9.02, 95%CI 4.81–16.92), having a parent with stomach cancer (OR3.93, 95%CI 2.17–7.14), having dyspepsia (OR 2.59, 95%CI 1.68–3.98), consumption of dried/salty food for the 1–2 and 3–4 times/week categories and all categories of fish consumption and in the protective effect seen with alcohol intake and in the overweight and obese categories of BMI. Elevated risks remained with diabetes and consumption of dried/salty food 5+ times/week, but were no longer statistically significant. The relationship between increasing quintiles of deprivation and stomach cancer risk was completely removed in the weighted analysis.

## Discussion

This study is the first national study of stomach cancer in an indigenous population and the first Māori-only population-based study of stomach cancer to be undertaken in NZ. We found sharing a room with >2 people as a child, being an ex-smoker, adult exposure to ETS, being tested for *H pylori*, having diabetes or dyspepsia, high intakes of salty food and fish and family history to be associated with stomach cancer. These results are consistent with those reported in other research while some established risk factors including alcohol and red meat intake and obesity were not found to be associated with stomach cancer in this study.

The increased risk seen with smoking and stomach cancer in this study is higher than that reported in a meta-analysis of cohort studies that compared former vs never smokers in males RR 1.42; 95%CI 1.21–1.67) [27] and a meta-analysis of 10,290 cases and 26,145 controls which reported a pooled OR of 1.12 (95%CI 0.99–1.27) for former and OR 1.25 (95%CI 1.11–1.40) for current smokers compared to never smokers [28]. We assessed the impact of SEP as a confounder on our results (S1 Table) which show a reduced OR in ex-smokers from 2.26 (95%CI 1.44–3.54) to 1.98 (95%CI 1.25–3.14) and in current smokers, from OR 1.46 (95% CI 0.86–2.48) to OR 1.15 (95%CI 0.67–1.99). Thus, there is evidence of some confounding by SEP, but our findings for ex-smokers are still higher than those published by Praud et al (28), and the confounding by SEP appears to be relatively weak. For current smokers, the situation is not so clear, since the OR changed more substantially after adjustment for SEP, though the adjusted estimate is still similar (but non-significant) to the estimate in the published meta-analysis (28). Māori smoking rates have reduced from about 60% in 1976 [29] but daily smoking rates in Māori remain almost three times those of European/Other (37.1% vs 13.6% respectively) [20]. The Māori smoking rate is similar to the highest rate reported in an OECD country (39% in Greece), whereas the average rate for OECD countries is 20% [38]. Māori also begin smoking at an earlier age than many other populations [30] when the sensitivity of adolescent lung tissue to smoking damage has been noted to have an independent effect on the development of lung cancer [39]. This, as well as, prolonged tobacco exposure at such high rates may also have implications for potential effects from ETS, which was associated with a significantly elevated risk of stomach cancer in the current study, similar to results found in some [40] but not all previous studies [41]. A tobacco control strategy with a strong emphasis on Māori-focused outcomes has been highlighted as a critical tobacco control activity for the NZ government to address inequalities and ensure a significant reduction in tobacco–related harm is achieved within the next decade [42].

The expected associations between alcohol intake [25] and obesity [19] and risk of stomach cancer were not seen in the current study. Instructions to report alcohol intake and height and weight one year previously were omitted in error on some of the case questionnaires and this created a problem with missing data for these variables; however, it is likely that any such missing data was missing at random, and therefore would not bias our results. In fact our results for diabetes are in accord with other studies on cancer risk and presence of diabetes mellitus [21], although the findings are more variable in relation to stomach cancer specifically [22–23]. The prevalence of diabetes is higher [20] and co-morbidity more common in Māori than European/Other [43–44]. This has important implications for cancer survival, given the generally poorer prognosis with stomach cancer, which, with the additional burden of co-morbidity, may further limit treatment offer or curative surgery [43]. The frequency distribution of BMI amongst controls in our study is consistent with those reported in other NZ literature [45].

More than two people sharing a sleeping space in childhood and being tested for *H pylori* were used as proxy measures for current or previous infection by this bacterium, with both factors associated with a significantly increased risk of stomach cancer. *H pylori* is an IARC Class 1 carcinogen [12], and considered a causative agent in the development of distal stomach cancer which accounts for 89% of gastric cancers worldwide [11]. The first NZ national estimate of the seroprevalence of *H pylori* infection found 35% seroprevalence in Māori compared to 18% in Europeans with ethnic differences in *H pylori* infection following patterns of household crowding by ethnicity [13–15]. Expanding measures to screen and treat those infected with *H pylori* alongside the provision of eradication therapy for asymptomatic infected persons are key population-level strategies that could reduce the incidence of stomach cancer in Māori [14, 46].

The joint presence of both smoking and *H pylori* exposure may be contributing to the significantly increased risks seen in this study where early life exposure to both risk factors are particularly relevant for Māori and has important implications for addressing the significant contribution that socio-economic factors have on health inequalities in NZ [47]. Although data is scarce, a positive interaction between *H pylori* infection and smoking on stomach cancer risk has been reported [48]. Our findings for dyspepsia are also relevant in this context given that gastritis is a precursor of gastric cancer [49] and that *H pylori* infection has been recently identified as the primary cause of chronic gastritis [50].

The strong associations found between stomach cancer risk and the highest categories of dried salty food and fish consumption are of interest. Salt-preserved foods have been consistently reported as a risk factor for stomach cancer [25–26] with processed meat classified as ‘carcinogenic to humans’ (Group 1) in respect of colorectal cancer and positively associated with stomach cancer [26]. The proportion of those consuming processed meat 1–2 times/week in the NZ general population [51] is very similar to our results (41.8% vs 43.4% respectively). Fish consumption has been more consistently reported as protective [25] rather than increasing risk, as was found in this study, while a recent meta-analysis concluded that the association between fish intake and gastric cancer remains unclear [52]. We did not find a protective effect with fruit and vegetable consumption on stomach cancer risk [24–25]. High anti-oxidant intake has been suggested to reduce the risk of gastric cancer particularly in long term smokers or those infected with *H pylori* where the gastric mucosa is more susceptible to oxidative stress [53].

Our findings are consistent with research showing an association between family history and stomach cancer [54]. Hereditary diffuse gastric cancer which is associated with germline E-cadherin gene alterations was first documented in Māori families in 1998 [10] however their contribution to the high rates of diffuse gastric cancer in Māori [8,9] remain unclear and will be the subject of future papers. The proportion of diffuse cancers in this study population is almost double that of the intestinal type. Diffuse type typically presents at a more advanced stage when prognosis is worse with survival differences documented between Māori and non-Māori [43–44]. This may have implications for current NZ standards of cancer service provision of first treatment within 62 days [55]. Elevated stomach cancer rates in indigenous compared to non-indigenous people have also been documented in the Circumpolar region, the US, Australia and Chile with increasing distal cancer rates noted as being particularly relevant given the relationship with SEP, household crowding and exposure to *H pylori* in these populations [56]. In our study, the highest proportion of cases was of the diffuse type (49.7%) which was also true for non-participating cases (35%). Additionally, case participants were more likely to have poorly differentiated (43% and 50% in non-participant cases) and distant spread tumours (36% and 60% in non-participant cases). A third of cancers were unstaged, possibly reflecting the difficulty in collecting this information due to the higher proportion of diffuse cancers. We also found lower rates of distal stomach cancer than reported in previous NZ studies [9]. Thus, future analyses of tumour site and histological type will be undertaken to further explore incidence patterns for proximal and distal stomach cancers as well as examine what may be influencing the high rates of diffuse type in Māori.

The study had several limitations, primarily the potential for selection and/or recall bias, which are common challenges in the conduct of case-control studies. The response rate for cases was relatively good (81%), but was only 37% for controls. Using information available on non-responders we were able to ascertain that response rates were lower amongst controls living in the most deprived quintile which may have introduced bias into our results in relation to those markers of SEP. To address the possibility of bias, we used a post-stratification weighting where the greatest effect was that seen for deprivation itself. For other factors, the weighting had the effect of diluting some (but not all) of the observed risks. Thus, given the potential for selection bias, we have been cautious in the interpretation of the results. The weighting does not adjust for differences between responders and non-responders in a given deprivation category, however there was little difference by gender or age between control responders and non-responders with the greatest difference being in Q5—hence weighting the results. Additionally, we were unable to adjust for exposure to *H pylori*, an important risk factor for development of distal stomach cancer but not for proximal stomach cancer (11,12). Our cases comprised a higher proportion of proximal rather than distal cancers; however, we cannot rule out the possibility that *H pylori* may be confounding some of the presented results. Importantly, there was also a high proportion of cases that had died or were too ill to take part in the study which would result in a potential underestimation of the impact of some of these risk factors on stomach cancer.

## Conclusion

Many of the findings in this, the first Māori-only population-based case control study of stomach cancer in Aotearoa/NZ, are in agreement with those previously reported in other countries. However, we have also identified some important differences in relation to established risk factors for stomach cancer. We emphasize the need to interpret the findings with care given the noted impact of selection bias on our results. While Māori continue to experience a markedly higher incidence of stomach cancer, this area of research must be considered a priority. Future emphasis is required on factors associated with elevated rates of diffuse stomach cancer, reducing inequalities by treating *H pylori* and further exploration of the contribution of smoking and ETS on stomach cancer risk.

## Supporting information

S1 File Stomach cancer study questionnaire. (PDF)

S2 File Study dataset. (XLS)

S1 Table Unweighted odds ratios (OR) a adjusted for deprivation and 95% confidence intervals (CI) showing the association between known risk factors and stomach cancer risk. ^a^ OR adjusted for gender, age and deprivation quintile,* Reference group, ** Number of people living in house divided by number of rooms, *** Environmental tobacco smoke.
(DOCX)
