# Supplementary material for: Risk of stomach cancer in Aotearoa/New Zealand: A Māori population based case-control study
Source: PLoS One. 2017 Jul 21;12(7):e0181581. doi: 10.1371/journal.pone.0181581 (PMC5521812; doi:10.1371/journal.pone.0181581)
Supplement: S1 File — (PDF) [file pone.0181581.s001.pdf]

Stomach Cancer

Study questionnaire

SECTION A: ABOUT YOU

In this first section, please tell us a few details about yourself

1) Before you start, please fill in today's date.

Day Month Year

2) What is your date of birth?

Day Month Year

3) **Were you born** in New Zealand?

Yes No Don't know

4) If you were not born in New Zealand, please tell us what age you were when you came to New Zealand

5) Which ethnic group do you belong to?
Tick the box or boxes which apply to you

New Zealand European 1

Māori 2

Samoan 3

Cook Island Māori 4

Tongan 5

Niuean 6

Chinese 7

Indian 8

Other (please specify) 9 ………………

Version 2: 01-08-08

SECTION B: ABOUT YOUR CHILDHOOD

We would like to get an idea of what your childhood was like. Please try to think back to what your life was like when you were <u>at primary school</u>. Do not worry if you cannot remember exactly.

Think about the house you lived in for the longest part of your childhood

6) How many rooms were there in that house?
<u>Please exclude</u> the hall, landing, kitchen, laundry / washroom, bathroom(s) and toilet(s)

☐ Rooms

7) How many people lived in that house?

☐ People

8) When you were **a child**, was either parent unemployed for any period of time?
Do not include working at home or housework as unemployment

Yes ☐ [1] No ☐ [2] I do not know ☐ [3]

9) When you were **a child**, did anyone in your household smoke?

☐ Yes ☐ No ☐ Don't know

10) When you were **a child**, how regularly did you eat fruit?

☐ [1] Every day
☐ [2] 5 to 6 days per week
☐ [3] 1 to 4 days per week
☐ [4] Less than once a week

11) When you were **a child**, how regularly did you eat vegetables (excluding potatoes)?

☐ [1] Every day
☐ [2] 5 to 6 days per week
☐ [3] 1 to 4 days per week
☐ [4] Less than once a week

12) Did your parents own or partly own their home (with or without a mortgage)

Yes ☐ [1] No ☐ [2] I do not know ☐

13) Did your parents own a car

Yes ☐ [1] No ☐ [2] I do not know ☐

14) Did your parents have a telephone in their house

Yes ☐ [1] No ☐ [2] I do not know ☐

SECTION C: ABOUT YOUR MOTHER

This section is about your mother.
Please answer the questions in relation to your biological mother.

15) Do you know, or know of, **your biological mother**?

Yes ☐ [1] No ☐ [2]

If **No,** go to **Section D**

If Yes,

16) What was your **mother's** date of birth?

☐ Day ☐ Month ☐ Year

If you only know the year, or month and year, please enter these

☐ ☐

17) Did (does) your **mother** suffer from any of the following conditions?
*Please tick either "**Yes**" or "**No**" for **each** condition*

i. Stomach cancer	Yes [1] ☐	No [2] ☐
ii. Breast Cancer	Yes [1] ☐	No [2] ☐
iii. Bowel / Colon Cancer	Yes [1] ☐	No [2] ☐
iv. Ovarian Cancer	Yes [1] ☐	No [2] ☐
v. Lung Cancer	Yes [1] ☐	No [2] ☐
vi. Other Cancer (please specify below)	Yes [1] ☐	No [2] ☐

18) Which other cancer did (does) she have?

…………………………………………………………………………………

SECTION D: ABOUT YOUR FATHER

This section is about your father.
Please answer the questions in relation to your biological father.

19) Do you know, or know of, **your biological father**? Yes ☐ [1] No ☐ [2]

If **No,** go to **Section E**

If Yes,

20) What was your **father's** date of birth? ☐ Day ☐ Month ☐ Year

If you only know the year, or month and year, please enter these

☐ ☐

21) Did (does) your **father** suffer from any of the following conditions?
*Please tick either "**Yes**" or "**No**" for **each** condition*

i. Stomach cancer Yes [1] ☐ No [2] ☐

ii. Lung Cancer Yes [1] ☐ No [2] ☐

iii. Bowel / Colon Cancer Yes [1] ☐ No [2] ☐

iv. Prostate Cancer Yes [1] ☐ No [2] ☐

v. Other Cancer (please specify below) Yes [1] ☐ No [2] ☐

22) Which other cancer did (does) he have?

…………………………………………………………………………………

SECTION E: ABOUT YOUR BROTHERS AND SISTERS

In this section, we are interested in your brothers and sisters (siblings). In each of the following questions, please refer only to <u>full</u> siblings, i.e. those that have the same mother and father as you. Do not include half brothers or sisters, or adopted siblings.

23) How many **<u>full</u> brothers and sisters** do (did) you have?
If none, please enter "0" and go to ***Section F****.*
If any have died, please include them in this count.

i) Brothers ii) Sisters

24) How many **older** full brothers and sisters do (did) you have?
If you are the oldest, answer "0"
If you have one older brother/sister, answer "1" etc.
If you are one of a twin, please do not count <u>your</u> twin as older than you.
If you have older twin brothers/sisters, please count them separately.

i) Brothers ii) Sisters

25) Have any of your full brothers or sisters been diagnosed with any of the following conditions?
Please enter "0" if none of your siblings have had a particular condition.
If one sibling has had more than one condition, they may be counted more than once.

**Condition**	**Number of Brothers with Condition** A	**Number of Sisters with Condition** B
i. Stomach cancer		
ii. Lung Cancer		
iii. Breast Cancer		
iv. Prostate Cancer		N/A
v. Bowel / Colon Cancer		
vi. Other Cancer		

Please complete <u>each</u> box, with "0" or other appropriate number

SECTION F: YOUR LIFE-STYLE

In this section, we are interested in your life and life-style
Please answer the questions in reference to your life <u>one year ago</u>

26) In an average week **last year**, <u>how many times a week</u> did you exercise long enough to work up a sweat or so your heart beats rapidly?
*Please tick **<u>one</u>** box only*

None ☐ [1] 1-2 a week ☐ [2] 3-4 a week ☐ [3] 5+ a week ☐ [4]

27) How many servings of **vegetables** (excluding potatoes) did you usually eat each week? ☐ Servings

As a guide: a serving of vegetables is one handful <u>OR</u> 1/2 cup raw <u>OR</u> cooked <u>OR</u> 80g cooked

28) How many servings of **fruit** did you usually eat each week? ☐ Servings

As a guide: 1/2 cup cooked or raw fruit <u>OR</u> 120g <u>OR</u> one medium sized apple, pear or orange etc <u>OR</u> two smaller fruits e.g. plums, kiwifruit, feijoas etc

29) In an average week **last year**, <u>how many times a week</u> did you eat **red** meat? (e.g. beef, lamb. Include mince, meat in pies etc)
*Please tick **<u>one</u>** box only*

None ☐ [1] 1-2 a week ☐ [2] 3-4 a week ☐ [3] 5+ a week ☐ [4]

30) In an average week **last year**, <u>how many times a week</u> did you eat **white** meat? (e.g. chicken, pork. Include mince, meat in pies etc)
*Please tick **<u>one</u>** box only*

None ☐ [1] 1-2 a week ☐ [2] 3-4 a week ☐ [3] 5+ a week ☐ [4]

31) In an average week **last year**, <u>how many times a week</u> did you eat **fish**? (Include shellfish)
*Please tick **<u>one</u>** box only*

None ☐ [1] 1-2 a week ☐ [2] 3-4 a week ☐ [3] 5+ a week ☐ [4]

32) In an average week **last year**, how many times a week did you eat **dried or salted food**? (hams, bacon, salami)
*Please tick **one** box only*

None ☐ [1] 1-2 a week ☐ [2] 3-4 a week ☐ [3] 5+ a week ☐ [4]

SMOKING

33) During your adult life, has anyone you live with ever smoked regularly? Yes ☐ [1] No ☐ [2]

34) Have you **ever** smoked, now or in the past? Yes ☐ [1] No ☐ [2]

If **No**, go to **Question 42**

35) What **age** were you when you started smoking regularly? ☐ Years

36) Are you a **current** smoker? Yes ☐ [1] No ☐ [2]

If **No**, go to **Question 40**

CURRENT SMOKERS ONLY

37) **If Yes,** on average, how many cigarettes do you smoke in a day?
*Please tick **one** box only*

Under 10 a day ☐ [4] 10 to 19 a day ☐ [2] 20 or more a day ☐ [3]

PAST SMOKERS ONLY

38) On average, how many cigarettes did you used to smoke in a day?
*Please tick **one** box only*

Under 10 a day ☐ [4] 10 to 19 a day ☐ [2] 20 or more a day ☐ [3]

39) How old were you when you STOPPED smoking? ☐ Years

ALCOHOL

The following questions are about drinking alcohol. As a guide, a drink is: a can or small bottle of beer (a third of a pub jug) OR a small glass of wine OR a nip of spirits (a 'single' in a pub)

40) **On average last year,** how often did you have a drink containing alcohol?
*Please tick **one** box only*

1 ☐	2 ☐	3 ☐	4 ☐	5 ☐
Never	Monthly or less	2 to 4 times a month	2 to 3 times a week	4 or more times a week

41) **On average last year,** how many drinks containing alcohol did you have on a typical day when you are drinking?
*Please tick **one** box only*

1 ☐	2 ☐	3 ☐	4 ☐	5 ☐
1 or 2	3 or 4	5 or 6	7 to 9	10 or more

42) What **age** were you when you started drinking alcohol regularly? ☐ Years

SECTION G: YOUR EARLIER LIFE-STYLE

We are interested in your life-style when you were younger. Please answer the questions in reference to your life at ages 20 years and 40 years. If you are younger than 40, please leave these boxes blank.

WHEN YOU WERE 20 YEARS OLD:

43) How often did you have a drink containing alcohol? *Please tick **one** box*

- 1 ☐ Never
- 2 ☐ Monthly or less
- 3 ☐ 2 to 4 times a month
- 4 ☐ 2 to 3 times a week
- 5 ☐ 4 or more times a week

44) How many drinks containing alcohol did you have on a typical day when you are drinking? *Please tick **one** box only*

- 1 ☐ 1 or 2
- 2 ☐ 3 or 4
- 3 ☐ 5 or 6
- 4 ☐ 7 to 9
- 5 ☐ 10 or more

45) At the **age of 20**, which of the following best described you? *Please tick **one** box only*

- 1 ☐ Not at all physically active
- 2 ☐ Not very physically active
- 3 ☐ Fairly physically active
- 4 ☐ Very physically active

WHEN YOU WERE 40 YEARS OLD:

46) How often did you have a drink containing alcohol? *Please tick **one** box*

- 1 ☐ Never
- 2 ☐ Monthly or less
- 3 ☐ 2 to 4 times a month
- 4 ☐ 2 to 3 times a week
- 5 ☐ 4 or more times a week

47) How many drinks containing alcohol did you have on a typical day when you are drinking? *Please tick **one** box only*

- 1 ☐ 1 or 2
- 2 ☐ 3 or 4
- 3 ☐ 5 or 6
- 4 ☐ 7 to 9
- 5 ☐ 10 or more

48) At the **age of 40**, which of the following best described you? *Please tick **one** box only*

- 1 ☐ Not at all physically active
- 2 ☐ Not very physically active
- 3 ☐ Fairly physically active
- 4 ☐ Very physically active

SECTION H: YOUR WORK

49) Please complete the table below with regards to work that you have ever done.
List **all** the jobs you have held in order from the first job you ever held. Please include paid and unpaid work
The first line is an example

	Who was your **employer**? (Name and Location)	**WHEN** did you work for this employer? FROM	TO	What was the **main activity** of the organisation?	What was your **department?**	What was your **job title?**
**Example**	J.B.Manufacturers, Hamilton	04/1975	08/1994	Manufacturing picture frame mouldings	Office	Administrator
**1.**						
**2.**						
**3.**						
**4.**						
**5.**						
**6.**						

Please use an additional sheet of paper if necessary.

SECTION I: YOUR HEALTH

50) Do you have a health practitioner or service for example, a doctor or nurse or other service that you usually **first** go to see when you are feeling unwell or are injured?

☐ Yes ☐ No ☐ Don't know

What sort of practitioner is this?

- My Family Doctor or regular GP ☐ 1
- The most convenient GP at the time ☐ 2
- The Accident and Emergency at a public hospital ☐ 3
- The Accident and Emergency at a private clinic ☐ 4
- Maori /Pacific health provider ☐ 5
- Community-based health provider ☐ 6
- Other *please specify* ☐ 7

51) What is your height in centimetres or feet/inches? **(One year ago)** ☐

52) What is your weight in kilograms or stones/pounds? **(One year ago)** ☐

DIABETES

53) Have you ever been told by a doctor that you have diabetes or sugar in the blood?

Yes ☐ 1 No ☐ 2

If **No**, go to **Question 59**

If Yes,

54) In what year was your diabetes first diagnosed? ☐

55) In what year did you begin regular treatment (with diet, tablets or injections) for your diabetes? ☐

56) Are you on regular treatment with <u>insulin</u> for your diabetes? Yes ☐ [1] No ☐ [2]

57) Are you on regular <u>tablets</u> for your diabetes? Yes ☐ [1] No ☐ [2]

If **No**, go to **Question 59**

If Yes,

58) Please give the name of medication

....

CANCER

59) Have you ever been told by a doctor that you have cancer? Yes ☐ [1] No ☐ [2]

If **No**, go to **Question 62**

If Yes,

60) In what year was your cancer first diagnosed? ☐

61) What sort of cancer is it?

....

H pylori

62) Have you ever been treated for a stomach bug (*H Pylori*) that required antibiotics and anti-ulcer drugs? (e.g Losec) Yes ☐ [1] No ☐ [2]

If **No**, go to **Question 65**

If Yes,

63) In what year were you tested? ☐

64) What treatment did you receive?

....

DYSPESIA

65) Have you ever been told by a doctor that you have heartburn (a burning pain behind the breastbone)? (this is also known as dyspepsia) Yes ☐ [1] No ☐ [2]

If **No**, go to **Question 67**

If Yes,

66) In what year was your heartburn first diagnosed? ☐

MEDICATION

67) Have you ever been treated with the following medications:

H2 blockers (reduce the amount of acid produced by the stomach e.g Tagamet, Zantac, Pepcid) — Yes 1 ☐ No 2 ☐

How long were you treated for: From ☐ To ☐

Proton pump inhibitors (reduce the amount of acid produced by the stomach e.g Aciphex, Nexium, Prevacid, Prilosec) — Yes 1 ☐ No 2 ☐

How long were you treated for: From ☐ To ☐

NOTE THIS SECTION IS INCLUDED IN THE QUESTIONNAIRE FOR CASES ONLY

We realise that you have recently been diagnosed with cancer, and would like to know more about what treatment you have received. Please complete the following questions, which will be treated with utmost confidentiality.

68) How was your stomach cancer detected?.

- Through symptoms 3 ☐
- Through GP 4 ☐
- Other *please specify* 5 ☐ …………………

69) In relation to your stomach cancer, when did your first notice signs or symptoms? *Give month and year, eg 08/2001*

☐ Month ☐ Year

70) When did you visit your GP regarding symptoms? *Give month and year, eg 08/2001*

☐ Month ☐ Year

71) After visiting your GP how long did you have to wait before your first appointment with a hospital doctor/specialist?

1 ☐	2 ☐	3 ☐	4 ☐	5 ☐
Same or next day	More than 2 days – 1 week	More than 1 week – 2 weeks	More than 2 weeks – 1 month	More than 1 month – 3 months

72) When did you first see a hospital doctor/specialist? *Give month and year, eg 08/2001*

☐ Month ☐ Year

73) Were you told what was wrong with you during this first hospital/specialist appointment, or was it before or after this first hospital/specialist appointment?

- Before 1st appointment ☐ 1
- At 1st appointment ☐ 2
- After 1st appointment ☐ 3

74) If you were told after this appointment, then how long after?
(*If you were told at this appointment or before, please go to Question 75)*

1 day – 2 weeks ☐ 1
More than 2 weeks – 1 month ☐ 2
More than 1 month – 3 months ☐ 3
More than 3-Months – 6 months ☐ 4
More than 6 months ☐ 5
Not been told ☐ 6

75) In relation to your stomach cancer care, has the hospital or clinic changed any of your appointments?

Yes ☐ 1 No ☐ 2

If so, how many? ☐

76) In relation to your stomach cancer care have you ever had to miss any appointments?

Yes ☐ 1 No ☐ 2

Specify circumstances:

……………………………………………………………………………………

……………………………………………………………………………………

77) What treatment did you receive?
Do not worry if you do not know

i)	Radiotherapy (X-rays)	Yes ☐ 1	No ☐ 2	Don't know ☐ 3
ii)	Chemotherapy	Yes ☐ 1	No ☐ 2	Don't know ☐ 3
iii)	Surgery	Yes ☐ 1	No ☐ 2	Don't know ☐ 3

78) Where did you have your treatment?

……………………………………………………………………………………

We would like to get further information about your treatment.

79) Can we ask your doctor(s) for copies of your medical records relating to your stomach cancer?

Yes ☐ [1] No ☐ [2]

Please note that if you answered NO to the above question, we will not approach your doctors.

****

Thank you <u>very much</u> for taking the time to complete this questionnaire. Your help assists us enormously in our research.

We will send you a copy of the study results when the research is completed.

Name:...

Address: ....

....

We may wish to involve you in further studies in the future. Please indicate your preference below regarding this.

Yes, I am happy to be contacted for future research ☐

OR

No, I do not want to be contacted for future research ☐
