# Supplementary material for: Risk of stomach cancer in Aotearoa/New Zealand: A Māori population based case-control study
Source: PLoS One. 2017 Jul 21;12(7):e0181581. doi: 10.1371/journal.pone.0181581 (PMC5521812; doi:10.1371/journal.pone.0181581)
Supplement: S1 Table — a OR adjusted for gender, age and deprivation quintile,* Reference group, ** Number of people living in house divided by number of rooms, *** Environmental tobacco smoke. (DOCX) [file pone.0181581.s003.docx]

**S1 Table. Unweighted odds ratios (OR) ^a^ adjusted for deprivation and 95% confidence intervals (CI) showing the association between known risk factors and stomach cancer risk**

|  | **OR [95% CI]^a^** |
| --- | --- |
| **Demographics** |  |
| **Childhood people to room ratio**** |  |
| <=1.0 | 1* |
| >1.0–2.0 | 1.42 [0.88-2.3] |
| >2.0 | 2.55 [1.48-4.42] |
| **Parent with stomach cancer** |  |
| No | 1* |
| Yes | 4.22 [2.23-7.98] |
| **Lifestyle factors** |  |
| **Smoking status** |  |
| Non-smoker | 1* |
| Ex-smoker | 1.98 [1.25-3.14] |
| Current smoker | 1.15 [0.67-1.99] |
| **Exposure to ETS*** as an adult** |  |
| No | 1* |
| Yes | 2.84 [1.66-4.87] |
| **BMI (kg/m^2^)** |  |
| <25 | 1* |
| 25-30 | 0.67 [0.43-1.05] |
| >30 | 0.3 [0.19-0.49] |
| **In the past year, how many times/week did you exercise?** |  |
| None | 1* |
| 1-2 | 0.64 [0.34-1.18] |
| 3-4 | 1.28 [0.75-2.19] |
| 5+ / week | 1.06 [0.65-1.74] |
| **In the past year, how often did you have drink containing alcohol?** |  |
| Never | 1* |
| Monthly or less | 0.94 [0.60-1.48] |
| 2 to 4 / month | 0.34 [0.18-0.65] |
| 2 to 3 / week | 0.27 [0.13-0.58] |
| 4 or + / week | 0.28 [0.13-0.57] |
| **Nutrition factors** |  |
| **In the past year, how many servings /week did you eat vegetables ?** |  |
| <7 | 0.28 [0.15-0.51] |
| 7-21 | 0.48 [0.31-0.74] |
| >= 21 / week | 1* |
| **In the past year, how many servings/week did you eat fruits?** |  |
| <7 | 0.51 [0.33-0.81] |
| 7-14 | 0.8 [0.52-1.25] |
| >=14 / week | 1* |
| **In the past year, how many times/week did you eat red meat?** |  |
| None | 1* |
| 1-2 | 0.59 [0.28-1.22] |
| 3-4 | 0.54 [0.26-1.08] |
| 5+ / week | 0.5 [0.23-1.08] |
| **In the past year, how many times/week did you eat white meat?** |  |
| None | 1* |
| 1-2 | 0.55 [0.25-1.24] |
| 3-4 | 0.91 [0.4-2.05] |
| 5+ / week | 0.48 [0.15-1.6] |
| **In the past year, how many times/week did you eat fish?** |  |
| None | 1* |
| 1-2 | 1.67 [1.02-2.71] |
| 3-4 | 4.25 [2.24-8.05] |
| 5+ / week | 3.74 [1.29-10.89] |
| **In the past year, how many times a week did you eat dried/salty food?** |  |
| None | 1* |
| 1-2 | 1.83 [1.22-2.73] |
| 3-4 | 4.99 [2.44-10.2] |
| 5+ / week | 2.45 [0.84-7.12] |
| **Other health factors** |  |
| **Ever diagnosed with diabetes** |  |
| No | *1 |
| Yes | 1.69 [1.08-2.64] |
| **Ever tested for *H pylori*** |  |
| No | *1 |
| Yes | 12.74 [6.26-25.91] |
| **Ever diagnosed with dyspepsia** |  |
| No | *1 |
| Yes | 2.55 [1.64-3.99] |

^a^ OR adjusted for gender, age and deprivation quintile

^*^ Reference group

^**^ Number of people living in house divided by number of rooms

^***^  Environmental tobacco smoke
